# Literary Note

**Published:** 1905-08

**Authors:** 


					﻿ITEM.
Japanese Post Cards in Battle.—A collector of post cards in
Saint Petersburg states, according to the Philadelphia Pithlic
Ledger, that all the soldiers in the Japanese army are supplied
with peculiar post cards. These cards are surrounded with
mourning border, printed on a piece of white silk, and are worn
by the Japanese soldiers on their chests. Before going to the
war they write on the post card the name and address of the
person to whom they wish the information in case of death to
be sent. In case of death on the field of battle the post cards
are stamped with the seal of the regiment, certifying the death of
the bearer, and are sent to Japan.
				

